# A molecular journey on the pathogenesis of primary hyperoxaluria

**DOI:** 10.1097/MNH.0000000000000987

**Published:** 2024-04-11

**Authors:** Barbara Cellini

**Affiliations:** Department of Medicine and Surgery, University of Perugia, Perugia, Italy

**Keywords:** kidney stones, pathogenic variant, primary hyperoxaluria, rare disorder

## Abstract

**Purpose of review:**

Primary hyperoxalurias (PHs) are rare disorders caused by the deficit of liver enzymes involved in glyoxylate metabolism. Their main hallmark is the increased excretion of oxalate leading to the deposition of calcium oxalate stones in the urinary tract. This review describes the molecular aspects of PHs and their relevance for the clinical management of patients.

**Recent findings:**

Recently, the study of PHs pathogenesis has received great attention. The development of novel *in vitro* and *in vivo* models has allowed to elucidate how inherited mutations lead to enzyme deficit, as well as to confirm the pathogenicity of newly-identified mutations. In addition, a better knowledge of the metabolic consequences in disorders of liver glyoxylate detoxification has been crucial to identify the key players in liver oxalate production, thus leading to the identification and validation of new drug targets.

**Summary:**

The research on PHs at basic, translational and clinical level has improved our knowledge on the critical factors that modulate disease severity and the response to the available treatments, leading to the development of new drugs, either in preclinical stage or, very recently, approved for patient treatment.

## INTRODUCTION

About 7000 rare diseases are known so far, mostly caused by inherited mutations leading to a protein deficit. Since no treatment is available for the majority of them, the quality of life and the management of patients represent a significant burden for caregivers and health systems [1,2]. Although the ease of access to DNA sequencing has improved diagnosis, the interpretation of the effects of mutations on target proteins and of their pathological relevance still represents an issue. In addition, even when the inheritance pattern is defined, other aspects of pathogenesis can remain unclear, including genotype/phenotype correlations in terms of disease severity and treatment responsiveness, or the metabolic effects of the protein deficit. The latter aspects assume particular relevance for enzyme deficits.

Suitable models should be employed to study the molecular pathogenesis of rare diseases, ranging from (i) *in silico* simulations, (ii) molecular analyses on purified proteins, (iii) models based on either cell lines, stem cells, or patients-derived cells, and (iv) animal models. A perfect model should mimic the protein deficit by reproducing an environment similar to that of the organ(s) affected in patients. This is not an easy task to achieve, and the integration of different methodologies is often necessary to understand the effects of mutations and their possible cross-talk, predict the response to treatment and discover new treatment strategies.

Primary hyperoxalurias (PHs) are a group of renal diseases due to the deficit of enzymes involved in the hepatic metabolism of glyoxylate. Glyoxylate is the precursor of oxalate, an end-product of metabolism excreted by urine [3]. The main hallmark of PHs is the increased urinary excretion of oxalate that precipitates as calcium oxalate stones leading to urolithiasis and nephrocalcinosis, eventually causing renal failure and progressing to systemic oxalosis [4]. This review provides an up-to-date summary on the molecular pathogenesis of PHs, whose investigation has been extremely flourishing in the last years, driven by increased disease awareness, easier access to genetic diagnosis, and availability of new therapeutics [5,6]. 

**Box 1:**
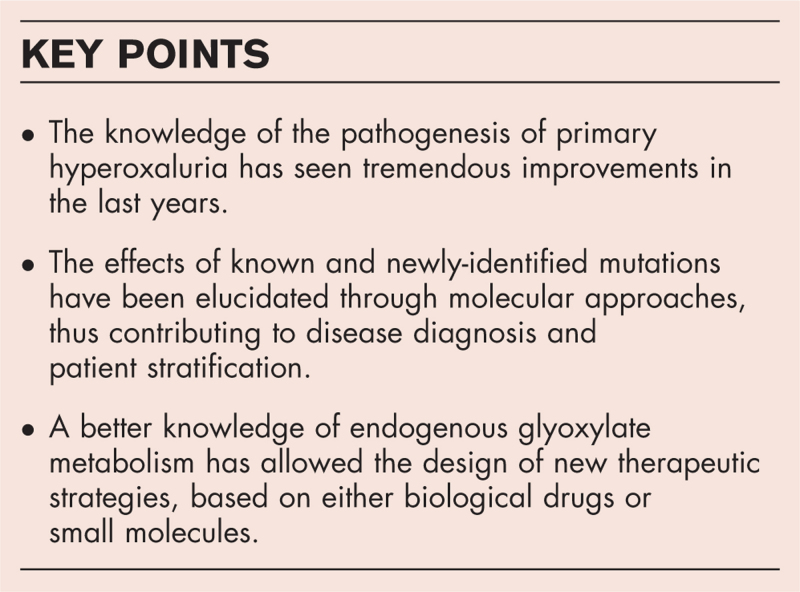
no caption available

## PRIMARY HYPEROXALURIAS ARE DISORDERS OF GLYOXYLATE METABOLISM

**FIGURE 1:**
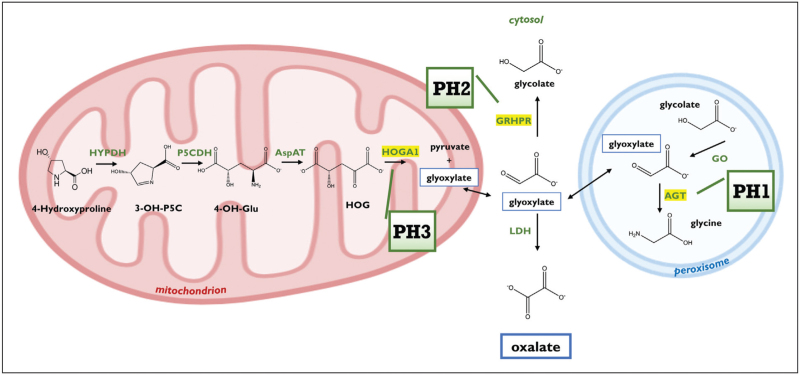
Molecular pathogenesis of PH. The main pathways of liver glyoxylate metabolism are shown highlighting the enzymes involved in the three forms of PH. See text for details. AGT, alanine:glyoxylate aminotransferase; GRHPR, glyoxylate/hydroxypyruvate reductase; HOGA1, 4-hydroxy-2-oxoglutarate aldolase; LDH, lactate dehydrogenase; 3-OH-P5C, 3-hydroxy-pyrroline 5-carboxylate; 4-OH-Glu, 4-hydroxyglutamate; HOG, 4-hydroxy-2-oxoglutarate; PH, primary hyperoxaluria.

The three forms of PH characterized until now, named PH1, PH2 and PH3, are due to the deficit of liver enzymes involved in the metabolism of glyoxylate, whose accumulation generates oxalate through the action of lactate dehydrogenase (LDH) (Fig. 1) [7,8]. PH1 (OMIM259900), the most frequent and severe form, is caused by the deficit of alanine:glyoxylate aminotransferase (AGT), a Vitamin B6-dependent peroxisomal enzyme that catalyzes a transaminase reaction converting l-alanine and glyoxylate to pyruvate and glycine, respectively [9,10]. In PH1 patients, peroxisomal glyoxylate generated from glycolate through glycolate oxidase (GO) is not detoxified by AGT, accumulates in the cytosol where it is oxidized to oxalate by LDH. PH2 (OMIM 260000) is caused by the deficit of glyoxylate/hydroxypyruvate reductase (GRHPR), which reduces glyoxylate to glycolate in cytosol and mitochondria, thus preventing its competing oxidation [11–13]. PH3 (OMIM 613616) is caused by mutations that functionally inactivate 4-hydroxy-2-oxoglutarate aldolase (HOGA1), which metabolizes 4-hydroxy-2-oxoglutarate (HOG) to give glyoxylate and pyruvate [14,15].

## MOLECULAR PATHOGENESIS OF PRIMARY HYPEROXALURIA TYPE 1 (PH1)

Human AGT localizes in the peroxisomal matrix thanks to a C-terminal targeting sequence, and utilizes pyridoxal phosphate (PLP) as coenzyme [10]. The protein forms a tight homodimer through a prevalently hydrophobic interface [16], as well as an N-terminal arm wrapping over the neighboring subunit, which strongly influences proper folding [17,18]. The equilibrium constant of transamination is largely shifted toward glyoxylate-to-glycine conversion, a peculiar property related to the physiological role of the enzyme [10]. The discovery of *AGXT* encoding AGT as the gene involved in PH1, which dates back to 1986 [9], has laid the foundations for the introduction of two therapeutic approaches which remained the only ones available until a few years ago: (i) the administration of Vitamin B6, a PLP precursor, which promotes the formation of the holoenzyme [19,20], and (ii) liver transplantation, that restores glyoxylate detoxification by substituting the entire pool of AGT. Both approaches present major shortcomings as Vitamin B6 is poorly effective, while liver transplant comes with numerous side-effects [21^▪^]. In the last decade, the in-depth analysis of the pathways generating endogenous oxalate have led to the approval of two novel drugs based on RNA silencing that target either GO or LDH [22,23^▪▪^,^24^^▪▪^]. Moreover, they prompted for translational research studies on the use of DNA editing [25^▪▪^–27^▪▪^] or small molecules behaving as GO and/or LDH inhibitors currently tested in preclinical models [28,29^▪^,^30^–^32^,33^▪^].

Although the new therapies have dramatically improved the prognosis of PH1 patients, their high costs and the individual different responsiveness [34] have highlighted the problem of correct diagnosis and patient stratification [35–37]. Indeed, the molecular pathogenesis of PH1 is quite complex. More than 300 mutations are currently listed in the ClinVar database and most of them are missense changes involving residues spread over the enzyme structure. A basic classification of the defects of the AGT variants, based on studies in both purified proteins and cellular models, distinguishes two main categories [5,38]. The first comprises variants displaying catalytic defects, characterized by mutations of residues critical for coenzyme/substrate binding or involved in the transamination reaction pathway (e.g. Gly82, Trp108 or Asp187). The second includes variants showing folding defects, which retain a biologically meaningful catalytic activity but are endowed with structural alterations affecting their folding efficiency, so that the levels of functional protein in the cell are strongly reduced. Most PH1-associated mutations belong to the second group, and lead to a variety of downstream effects, including an increased tendency to aggregation or intracellular degradation, or the mistargeting of AGT to mitochondria where the protein is metabolically ineffective.

**FIGURE 2:**
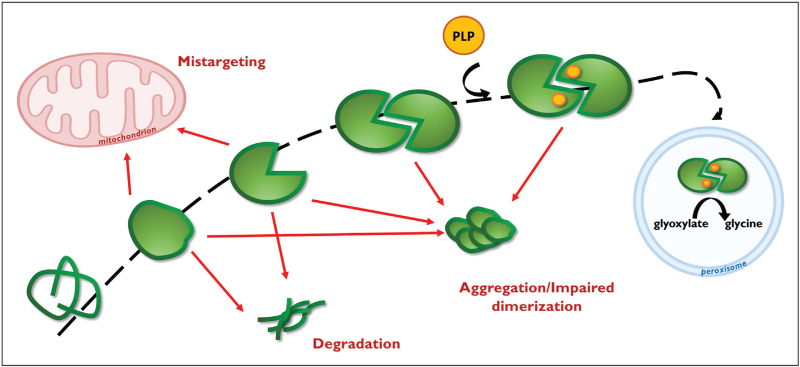
AGT folding pathway and effect of PH1-associated mutations. U, unfolded monomer; M∗, partially folded monomer; M, folded monomer; D, apodimer; DPLP, PH, primary hyperoxaluria; PLP-bound dimer. See the text for details.

The realization that PH1 is mostly a misfolding disease has paved the way for a re-evaluation of the therapy with Vitamin B6. Indeed, PLP is not only essential as prosthetic group in the catalytic pathway, but also plays a crucial role in promoting the correct folding of AGT. As depicted in Fig. 2, AGT is synthetized in the cytosol as unfolded chain (U) that probably generates a partly-folded monomer (M∗) which then dimerizes (D) and binds PLP (D_PLP_). The fully-folded D_PLP_ form is then kept by the peroxisomal import machinery and imported in the peroxisomal matrix [39]. Through binding at the active site in a cleft formed by residues coming from both subunits, PLP promotes AGT dimerization thus shifting the equilibrium toward the native structure that is functional, stable and correctly localized [40]. As such, it behaves as a chaperone for variants showing structural defects because it thermodynamically stabilizes the dimer reducing the amounts of monomeric forms that are prone to degradation, aggregation, and aberrant import [16]. This explains the responsiveness of some conformational variants to Vitamin B6 at clinical level, for example, p.Gly170Arg, p.Phe152Ile, and p.Gly41Arg, or in *in vitro* studies, for example p.Gly47Arg, p.Ile56Asn [41,42^▪^]. The role of misfolding in PH1 pathogenesis has also prompted the identification of small AGT ligands working as pharmacological chaperones for the holo-form, that is, competitive inhibitors that stabilize the native structure and dissociate in the presence of the substrate [5]. A recent study has identified a hit compound as putative drug able to partly rescue the effect of the most common pathogenic mutations in a cellular model of PH1 [43,44].

An important aspect contributing to PH1 pathogenesis is that the *AGXT* gene comes in two allelic forms, i.e. a more frequent major allele and a polymorphic minor allele, characterized by a protein product (AGT-Mi) with p.Pro11Leu and p.Ile340Met substitutions. AGT-Mi is not pathogenic, but shows a lower stability as compared with the major allelic form. Some mutations are pathogenic only when inherited on the background of the minor allele [38,45–47]. Indeed, the mutation of Pro11 in AGT-Mi generates a putative mitochondrial targeting sequence that is not effective unless another mutation (as the p.Gly170Arg) causes a destabilization of the dimeric structure that is sufficient to redirect the protein to the wrong compartment. Other mutations are pathogenic on both allelic backgrounds, but the enzymatic and clinical phenotype is more severe on the minor allele, also affecting the response to Vitamin B6 [42^▪^,^48^,^49^]. Structural studies have provided evidence for an increased plasticity of the minor allelic form as compared with the major one, thus allowing to explain the molecular reasons behind the synergistic effect of polymorphic and pathogenic mutations [50^▪^,51^▪^].

The relation between the molecular and clinical aspects of PH1 can be exploited not only to explain or predict treatment responsiveness, but also to interpret the effects of newly-identified mutations. In the last years, a more rapid and comprehensive genetic characterization of hyperoxaluria patients has been performed, which in turn has increased the number of new mutations identified in the genes associated with the three forms of PH [52–54,55^▪^,56^▪^]. This situation has posed new challenges in the interpretation of missense variants, and has highlighted the use of functional studies to support pathogenicity assessment [57]. As for PH1, our group recently setup a new cellular model of disease based on HepG2 cells, a cell line of hepatic origin showing a conserved glyoxylate/oxalate metabolism [58,59]. The model was used to support diagnosis in a PH1 patient who was compound heterozygous for a validated pathogenic mutation and a novel mutation, p.Gly365Cys [60^▪^]. Through functional studies at protein and cellular level, we confirmed the pathogenicity of the Gly365 mutation by showing that it induces a conformational change that compromises PLP binding, in turn causing a catalytic impairment and a reduced intracellular stability of AGT.

## MOLECULAR PATHOGENESIS OF PRIMARY HYPEROXALURIA TYPE 2 (PH2)

PH2 is a very rare form of PH due to the deficit of GRHPR, a homodimeric enzyme that catalyzes the NADPH-dependent reduction of both glyoxylate and hydroxypyruvate generating glycolate and D-glycerate, respectively [61]. PH2 can display a morbidity higher than expected [12,62], which warrants a more in-depth characterization of its pathogenetic mechanisms. In this regard, it has been demonstrated that GRHPR shows a double cytosolic/mitochondrial localization, thus expanding its role in mitochondrial glyoxylate detoxification as claimed from stable isotope infusion studies [13,63].

No specific therapies are available for PH2 except for conservative treatments, although the high liver expression of GRHPR has supported the introduction of liver transplantation [64,65]. The use of siRNA-drugs against LDH has been also evaluated, due to its presumed involvement in oxalate generation in PH2 patients. However, the variable results obtained from clinical studies [66] warrant a more detailed examination of liver glyoxylate metabolism in PH2 as well as the possible role of other organs in the pathogenesis of the disease [63].

ClinVar lists more than 100 mutations on the *GRHPR* gene as pathogenic or likely pathogenic, but the limited number of patients does not allow to define genotype/phenotype correlations [11]. Most mutations are frameshift or null, with straightforward effects. The studies on missense mutations agree upon the finding that altered coenzyme binding and/or a compromised catalysis are the main defects driving GRHPR loss-of-function in PH2, while folding defects are less common [67].

In the future, we expect a better knowledge of the molecular mechanisms of PH2, which could lay the foundations for the identification of pharmacological targets and the development of specific therapies. In this regard, the finding that hydroxyproline is the major source of oxalate in PH2 patients [63], has prompted the targeting of enzymes involved in hydroxyproline catabolism. In particular, hydroxyproline dehydrogenase has been validated *in vivo*[68], and substrate analogues acting as inhibitors have been identified, an important step toward the development of a new pharmacological treatment [69].

## MOLECULAR PATHOGENESIS OF PRIMARY HYPEROXALURIA TYPE 3 (PH3)

PH3 is considered the mildest form of PH, because most patients have a preserved renal function and, although the symptoms present early in infancy, they tend to disappear in adulthood [70]. Nonetheless, recent studies suggest that the severity of the disease could be higher than expected [71]. The genetic causes of PH3 are mutations in the *HOGA1* gene encoding HOGA1, a mitochondrial enzyme involved in the last step of hydroxyproline catabolism [14]. HOGA1 is a type I aldolase that cleaves HOG generating pyruvate and glyoxylate [72]. Disease-associated mutations prevent HOGA1 functionality, leading to the accumulation of HOG and its by-products such as 4-hydroxyglutamate and 2,4-dihydroxyglutarate [73].

PH3 accounts for approximately 10% of PH cases, but carrier frequencies suggest that the true prevalence is probably underestimated [74]. Moreover, heterozygosity for mutations in the *HOGA1* gene could be a risk factor for calcium oxalate urolithiasis [75]. These findings highlight that the disease is probably underdiagnosed and that a better knowledge of its pathogenesis could benefit a number of patients affected by kidney stones. Among the >400 mutations on the *HOGA1* gene listed in ClinVar, 88 are classified as pathogenic, including 43 missense changes. The most common are p.Glu315del and the intronic insertion p.700+5G>T leading to a C-terminal insertion of a stretch of 17 amino acids [74]. Only few studies have been specifically addressed to analyze the molecular effects of disease-causing mutations in HOGA1. Based on the enzyme structure, mutations that probably give rise to a catalytic defect have been identified, such as the p.Pro190Leu and p.Arg70Pro, which affect the positioning of residues critical for the reaction mechanism including Lys196 [72]. On the other hand, cellular models have unraveled the effects of mutations involving residues located far from the active site, including the two most common mutations that enhance tendency to degradation and/or aggregation [76,77]. A recent comprehensive *in silico* study on the effects of 57 HOGA1 mutations, indicates that most missense mutations are predicted to affect HOGA1 stability, although no specific genotype-phenotype correlations can be made [78^▪^].

Nonetheless, the main enigma on the molecular pathogenesis of PH3 is how the deficit of an enzyme involved in glyoxylate synthesis could lead to increased oxalate production. It is demonstrated that the deficit of HOGA1 causes the accumulation of the substrate HOG, as also observed in patients [79]. *In vivo* and *in vitro* studies have led to two hypotheses on accumulating HOG: it is transported to the cytosol where it is cleaved by a still unknown aldolase producing glyoxylate that then generates oxalate [80], or it inhibits GRHPR, thus preventing glyoxylate reduction to glycolate as occurs in PH2 [76,81]. A definitive proof is not currently available. Moreover, moving from the oxalate decarboxylase activity of HOGA1 that generates pyruvate, the possibility that the deficit promotes oxalate formation by altering mitochondrial redox balance has been proposed [82]. A more detailed knowledge of the metabolic role of HOGA1 in mitochondrial metabolism, coupled to the availability of suitable cellular and animal models of the disease, will lead to a better understanding of PH3 pathogenesis in the next future, with possible relapses toward the understanding of risk factors associated with kidney stones formation in general population.

## CONCLUSION

Discoveries on the alterations in endogenous metabolism of glyoxylate and oxalate in PH have vigorously improved the understanding of disease mechanisms and the development of new drugs. It is hoped that in the near future, thanks to the availability of more sophisticated models and analysis techniques, it will be possible to apply personalized medicine approaches in order to optimize resources and improve the management and quality of life of patients.

## Acknowledgements

*None.*

### Financial support and sponsorship

*This work was supported by Italian Ministry of University and Research (PRIN2022, 2022J7CKMJ) to B.C.*

### Conflicts of interest

*B.C. has received a grant from Dicerna Pharmaceuticals, a Novo-Nordisk subsidiary, Lexington, MA, USA.*

## REFERENCES AND RECOMMENDED READING

Papers of particular interest, published within the annual period of review, have been highlighted as:

▪ of special interest
▪▪ of outstanding interest
